# Differential Regional Immune Response in Chagas Disease

**DOI:** 10.1371/journal.pntd.0000417

**Published:** 2009-07-07

**Authors:** Juliana de Meis, Alexandre Morrot, Désio Aurélio Farias-de-Oliveira, Déa Maria Serra Villa-Verde, Wilson Savino

**Affiliations:** 1 Laboratory on Thymus Research, Oswaldo Cruz Institute, Oswaldo Cruz Foundation, Rio de Janeiro, Brazil; 2 Laboratory of Immunobiology, Institute of Microbiology Paulo de Góes, Federal University of Rio de Janeiro, Rio de Janeiro, Brazil; University of Edinburgh, United Kingdom

## Abstract

Following infection, lymphocytes expand exponentially and differentiate into effector cells to control infection and coordinate the multiple effector arms of the immune response. Soon after this expansion, the majority of antigen-specific lymphocytes die, thus keeping homeostasis, and a small pool of memory cells develops, providing long-term immunity to subsequent reinfection. The extent of infection and rate of pathogen clearance are thought to determine both the magnitude of cell expansion and the homeostatic contraction to a stable number of memory cells. This straight correlation between the kinetics of T cell response and the dynamics of lymphoid tissue cell numbers is a constant feature in acute infections yielded by pathogens that are cleared during the course of response. However, the regional dynamics of the immune response mounted against pathogens that are able to establish a persistent infection remain poorly understood. Herein we discuss the differential lymphocyte dynamics in distinct central and peripheral lymphoid organs following acute infection by *Trypanosoma cruzi*, the causative agent of Chagas disease. While the thymus and mesenteric lymph nodes undergo a severe atrophy with massive lymphocyte depletion, the spleen and subcutaneous lymph nodes expand due to T and B cell activation/proliferation. These events are regulated by cytokines, as well as parasite-derived moieties. In this regard, identifying the molecular mechanisms underlying regional lymphocyte dynamics secondary to *T. cruzi* infection may hopefully contribute to the design of novel immune intervention strategies to control pathology in this infection.

## Introduction

Chagas disease is caused by the protozoan *Trypanosoma cruzi*. The infection was initially rural in endemic areas in Latin America, transmitted by contaminated insect vectors of the family *Reduviidae*. Insects become vectors after biting *T. cruzi*-infected hosts (animals or humans). Parasites may also be transmitted by blood transfusion, by organ transplantation, orally, and congenitally. For this reason, Chagas disease is emerging in non-endemic countries such as Japan, Canada, Germany, Romania, Spain, and the United States [1],[2]. It is estimated that 14–16 million people in Latin America and 1 million in the US are infected with *T. cruzi* with 670,000 premature disabilities and deaths per year worldwide [1]–[3]. The infection is considered a world health problem and a neglected tropical disease with deficiencies in treatment, absence of appropriated vaccines, and world spreading [4],[5]. The complexity in treatment is related to the fact that current chemotherapic drugs Benznidazole and Nifurtimox are able to heal only a portion of recent infections, have severe side effects, and are active only in the acute phase and short-term chronic phase of infection [2].

Considered a “silent killer,” infection with *T. cruzi* leads to an acute phase, with symptoms such as fever, muscle pain, swollen lymph nodes, hepatosplenomegaly, pericardial effusion, and inflammatory reaction at the vector's biting site (chagoma) [2],[6]. During the acute phase, circulating parasites are numerous and able to infect several tissues in the host, including skeletal muscle, lymphoid tissues, nervous tissues, and glands [5],[7]. In humans, the acute phase is short (two months) and may lead to complications such as myocarditis or meningoencephalitis. Spontaneous recovery occurs in more than 95% of the patients [8]. Following the acute phase, the patient enters into a long indeterminate latent phase with no symptoms and very low parasitism. The latent infection remains silent for 10 to 30 years. About one third of infected patients in the latent phase develop clinical symptoms such as chronic cardiac dysfunction (cardiomyopathy), megacolon, or megaesophagus. The average life expectancy decreases about nine years in these clinical forms of chronic chagasic patients [8].

**Figure 1:**
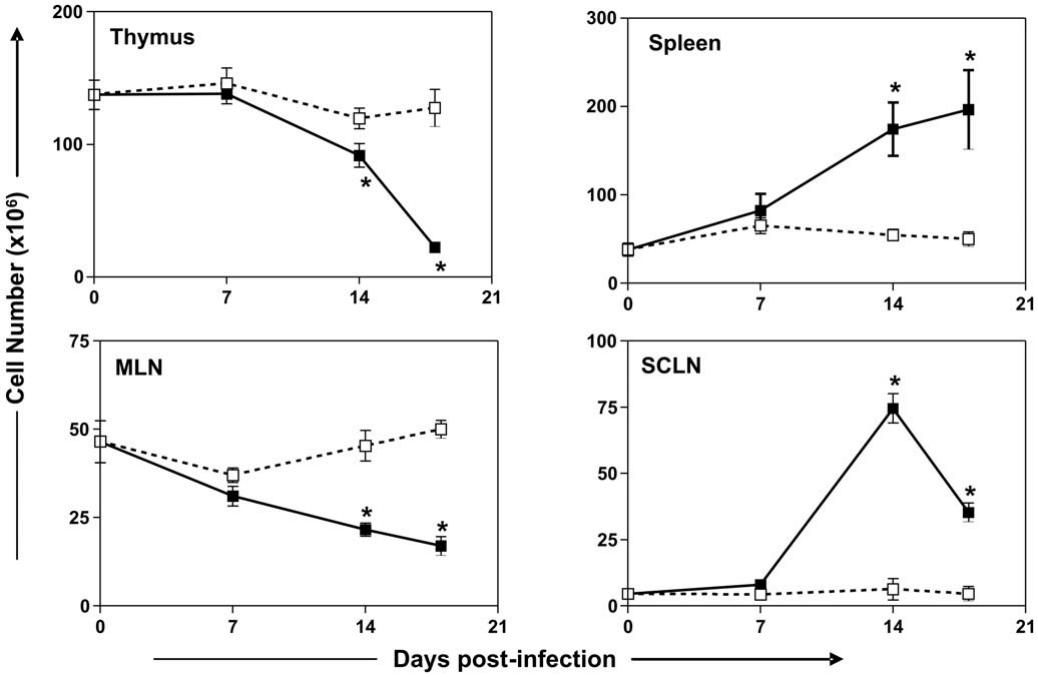
Differential fluctuations in the cellularity of the thymus, spleen, MLN, and SCLN in the course of acute *T. cruzi* infection. Note a lymphocyte expansion in the spleen and SCLN, in parallel to a lymphocyte decrease in the thymus and MLN. BALB/c mice were infected intraperitoneally with 10^2^ blood trypomastigotes of the *Tulahuén* strain, killed at different days of infection, and cell numbers evaluated by trypan blue exclusion. Erythrocytes were previously depleted in the spleen cell suspensions by treatment with Tris-buffered ammonium chloride. Values represent the mean±standard error; *n* = 3–5 mice/group in each point. Data recorded on thymus, spleen, MLN, and SCLN from *T. cruzi*-infected mice (closed squares) were compared to non-infected age-matched controls (open squares) with ANOVA statistical test, using the program SigmaStat (Statistical Software) for Windows. Data were considered significant if *p* values were <0.05. Data represent mean±standard error. All experiments and animal handling were conducted according to protocols approved by the Oswaldo Cruz Foundation Committee on the Use of Animals.

Experimental models of *T. cruzi* infection have been widely used to study various aspects of the infection, and the vast majority of knowledge of the biology of *T. cruzi* infection was first developed in the experimental mouse model. Acute infection in mice leads to strong activation of innate and adaptive immune response. Splenomegaly and expansion in subcutaneous lymph nodes (SCLN) were reported, with persistent T and B cell polyclonal activation (Figure 1) [9]–[11]. Conversely, atrophy in thymus and mesenteric lymph nodes (MLN) was also observed in the infection (Figure 1) [12]–[14].

Thus, it is conceivable that the immune response to *T. cruzi* infection is complex, and differential patterns of responses may occur in distinct compartments of the immune system, including cell expansion, cytokine production, and cell death. In this context, we review herein a number of findings showing that in the course of Chagas disease, the dynamics of lymphocyte response is diverse in distinct lymphoid tissues.

## Thymocyte Depletion and Differential Cytokine Profile in *T. cruzi* Infection

**Figure 2:**
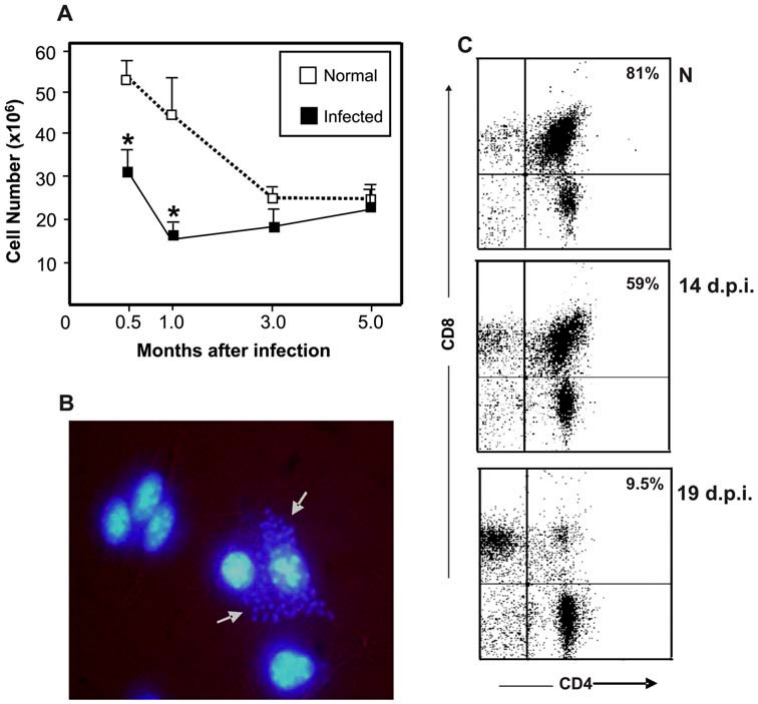
The thymus is a target organ in *T. cruzi* infection. Panel A shows the number of thymocytes in thymus of *T. cruzi*-infected mice (C3H/HeJ) during acute (one month) and chronic phases (after three to five months) of infection. Adapted from [32], *p<0.01. Panel B reveals the presence of the amastigote forms of the Colombian strain of *T. cruzi* within cultured thymic epithelial cells, ascertained by DAPI staining. Note that one cell (arrow) is deeply loaded with trypomastigote forms of the parasite in the cytoplasm. The mouse TEC line (IT-76M1) was cultured with 60 *T. cruzi* trypomastigotes/TEC for six hours; washed to remove free parasites, and cultured for a further 48 hours. Panel C shows a progressive thymocyte depletion in mice acutely infected with *T. cruzi*. BALB/c mice were infected intraperitoneally with 10^2^ blood-derived trypomastigotes of the *Tulahuén* strain, and killed at 14 and 19 days post-infection (d.p.i.). Percentage values of the CD4^+^CD8^+^ thymocytes and respective days post-infection are shown. In normal animals, the percentage of CD4^+^CD8^+^ thymocytes remained the same along with the experimental period.

The thymus is a primary lymphoid organ in which bone marrow-derived T cell precursors undergo differentiation, leading to migration of positively selected thymocytes to the T cell-dependent areas of secondary lymphoid organs [15]. This tissue is a target organ in *T. cruzi* infection in mice, where the parasite is able to infect thymic microenvironmental cells in vivo and in vitro [5],[12],[14] (Figure 2). Ultrastructural analysis of infected thymus indicates that phagocytic and epithelial cells can be infected in vivo [16].

Acute *T. cruzi* infection leads to thymus atrophy, with loss of thymus weight, decline in cell number, and depletion of CD4^+^CD8^+^ (DP) thymocytes [5], [14], [17]–[19]. Thymocyte depletion is detectable in early stages of infection and increases along with time until the peak of parasitemia (Figures 1 and 2). The loss of thymocytes observed in the acute phase of infection is apparently due to differences not only in cell death, but also in proliferation and migration of thymocytes.

Mitogenic responses of thymocytes from acutely infected mice is reduced due to decrease in interleukin (IL)-2 production, which in turn is associated with high levels of IL-10 and interferon (IFN)-γ [20]. Additionally, increased production of IL-4, IL-5, and IL-6 was detected in thymocytes from acutely infected mice, being related to thymocyte cytotoxic activities [20].

Abnormal thymocyte migration is also observed in the thymus of infected mice. In vitro studies of thymocyte migration in thymic nurse cells (TNCs, specialized cortical thymic epithelial cells that harbor and release immature thymocytes as a consequence of cell migration) following in vivo and in vitro infection demonstrated that thymocytes from infected TNC complexes are released faster than the corresponding controls [5],[14],[21]. These studies suggested an increase in the migratory capacity of thymocytes from infected mice. In fact, the increase in DP cell migration from thymus to peripheral lymphoid organs seen in both acutely and chronically infected mice corroborates this hypothesis [14],[21],[22]. Phenotypic analysis of DP lymphocytes in SCLN from *T. cruzi*-infected BALB/c mice demonstrated that part of these cells expresses “forbidden” T cell receptors and high amounts of cell migration-related membrane receptors, including the integrins VLA-4, VLA-5 (fibronectin receptors), and VLA-6 (laminin receptors) [5],[21],[22].

Thymic microenvironmental cells apparently favor the abnormal thymocyte migration seen in infected mice. Several studies demonstrated that in these animals the thymus exhibits enhanced deposition of extracellular matrix proteins such as fibronectin, laminin, and type IV collagen, increased chemokine contents such as CXCL12, and expression of de-adhesive molecules such as galectin-3. Ex vivo cell migration experiments revealed that all these molecules would favor the abnormal release of DP thymocytes to peripheral lymphoid organs [5],[12],[21],[23],[24].

Independent research groups reported molecules involved in thymocyte death following experimental *T. cruzi* infection, comprising parasite- or host-derived factors. Examples of the participation of host-derived stimuli in thymocyte apoptosis are galectin-3, extracellular ATP, and glucocorticoid hormones [23], [25]–[27]. Interestingly, DP thymocyte depletion inside TNCs is dependent on androgens, and the intracellular thymocyte pathway of cell death leads to activation of caspase-3 [28]. Conversely, Fas and perforin are not involved in thymus atrophy in *T. cruzi* infection [19]. In relation to *T. cruzi*-derived molecules, it has been reported that the virulence factor trans-sialidase, an enzyme that alters cell sialylation, promotes apoptosis of DP thymocytes [29],[30].

Thymus alterations in *T. cruzi* infection occur concomitantly to the increasing parasitemia, suggesting that thymus atrophy is dependent on the parasite load [16],[21]. Corroborating this hypothesis, mice treated with benznidazole in the course of infection show reduction in blood parasitemia and no thymus alterations during infection [31]. Another interesting finding is that chronically infected mice do not exhibit significant reduction of thymus weight, cell number, and DP thymocytes, as compared with age-matched normal mice (Figure 2A) [32]. The similarity between normal and infected thymocyte cell number in the chronic phase might be related to the fact that in normal mice, thymocyte depletion is observed along with aging [33] (Figure 2A). The impact of thymus alterations during acute infection with respect to the development of effector immune response is still unknown. It is reasonable to think that a decline in the generation of T cells together with an abnormal release of non-selected thymocytes during acute infection would favor the parasite rather than the host. In fact, T lymphocytes are crucial for mounting an effective anti-*T. cruzi* immune response: athymic nude mice infected with *T. cruzi* show increased parasitemia and mortality rate and shortened survival time [16],[34]. On the other hand, it was previously suggested that thymus-derived TCRγδ T lymphocytes may have suppressor effects on the host immune response [35].

Taken together, these data support the idea that *T. cruzi* infection promotes disturbances of proliferation, migration, and cell death within the thymus.

## T Cell Depletion in Peyer's Patches and MLN Following *T. cruzi* Infection

The mucosal immune system remains less studied than it should be in *T. cruzi* infection. Such studies are necessary since: (1) the prognosis of the chronic phase depends on the evolution of the acute phase and the parasite is able to infect gut tissues; (2) chronic patients with Chagas disease may progress with digestive forms of clinical manifestations, namely megacolon and megaesophagus (in Brazil, the incidence of megaesophagus in endemic areas is over 8%); and (3) oral transmission of the parasite in humans through ingestion of fruit juice contaminated with *T. cruzi* recently occurred in Brazil, leading to a severe and sometimes lethal acute disease [36]–[39].

The Peyer's patches (PP) are important lymphoid organs implicated in mucosal immune response. PP are separated from the intestinal lumen by a layer of epithelial cells known as the follicle-associated epithelium, in which the so-called M cells are involved in mucosal immune responses, binding invasive pathogens and passing them to professional antigen-presenting cells inside PP. After encountering the antigen, antigen-presenting cells (mainly dendritic cells) migrate to T cell areas where they interact and activate T cells. Lymphocytes and dendritic cells that are primed in PP migrate to MLN through draining lymphatic vessels.

Considering that PP and MLN drain antigens from the small intestine and that chronic infection may progress with damage to the digestive tract, MLN and PP might have some relation to gut pathologies in infected patients. Very little information is available regarding the immunological response of chronic chagasic patients with gastrointestinal forms of the disease, and none of these works analyze gut-associated lymphoid tissues or MLN. It is known that patients with digestive forms of the disease present high parasitemia and decreased T/B lymphocyte numbers in their blood [40]. Moreover, peripheral blood mononuclear cells (PBMCs) from esophagopathy patients produce high levels of inflammatory cytokines such as IFN-γ and MIG and low levels of tumor necrosis factor (TNF)-α, with no significant differences in IL-4 and IL-5 production [41].

In experimental animal models, *T. cruzi* infection induces several forms of damage in gut-associated lymphoid tissues. PP from infected mice show reduction in size, number, and cellularity, due to an increase in T and B lymphocyte depletion [42]. MLN also undergo severe atrophy in acute infection in several models of *T. cruzi* infection [13],[43] (Figure 1). The diminished number of MLN lymphocytes in infected mice seems to be associated with differences in lymphocyte proliferation and death. MLN from *T. cruzi*-infected mice show reduced numbers of proliferating lymphocytes and decreased cytokine production (IL-2, IL-4, and IL-10) by activated T lymphocytes, as summarized in Table 1 [13],[44]. Interestingly, MLN T lymphocytes produce mainly type-1 cytokines (IFN-γ) upon infection with *T. cruzi* [44]. Increased lymphocyte death is also observed in MLN from *T. cruzi*-infected mice [13],[44].

**Table 1:** Differential cytokine and apoptosis/proliferation profiles in lymphoid organs of mice undergoing *T. cruzi* infection.

Response to Infection	Thymus	MLN	SCLN	Spleen	References
Apoptosis	↑↑	↑↑	↑	↑	[13],[23],[25],[44],[49],[51],[72],[73],[85]
Proliferation	↓	↓↓	↑↑	↑↑	[11],[13],[56],[57],[86]
IL-2	↓	↓	↑/ =	↓	[44], [65]–[67]
INF-γ	↑↑	↓	↑↑	↑↑	[20],[44],[49],[51],[65]
IL-4	↑↑	↓↓	↑↑	↑	[20],[44],[51],[52],[68]
IL-10	↑↑	↓↓	↑↑	↑↑	[20],[44],[51],[52],[68]

Differences between normal versus infected mice are indicated with the arrows.

T cell apoptosis can be stimulated in secondary lymphoid organs by activation-induced cell death (AICD) or growth factor withdrawal [45]. The abundance of antigens and cytokine production (IL-2 and IL-4) in the lymphoid microenvironment are essential to trigger the cell death pathway [45]. In the presence of an antigen, IL-2 prompts T cells to die by AICD, through activation of death receptor molecules and caspase-8 [46]–[48]. In the absence of antigen, deprivation of cytokines initiates the mitochondrial death pathway, promoting cytochrome *c* release into the cytoplasm and activating caspase-9 and downstream effector caspases [46]. In MLN from mice acutely infected with *T. cruzi*, T/B lymphocyte apoptosis occurs in early stages of infection through AICD and growth factor withdrawal mechanisms [13],[44]. Studies performed with the Colombian strain of *T. cruzi* in FasL mutant mice (*gld*) and TNF receptor-1 knockout mice suggested that these molecules are involved in MLN lymphocyte apoptosis from infected mice [13]. Moreover, acute infection with the Dm28c clone of the parasite led to IL-4 deprivation and caspase-9 activation, promoting MLN atrophy with T cell depletion [44].

Depletion of MLN lymphocytes impairs immune response against the parasite, since MLN excision prior to infection increases host susceptibility to infection [44]. Interestingly, in vivo administration of zVAD-fmk (a pan-caspase inhibitor) in the course of infection prevents MLN atrophy, reduces lymphocyte apoptosis in secondary lymphoid tissues, and increases host resistance to infection [44],[49]. Moreover, oral vaccination with *Salmonella enterica* carrying cruzipain showed protective immune response against the parasite with reduction of tissue damage of infected mice [50], thus showing that mucosa-associated lymphoid cells are important to host immunity in *T. cruzi* infection, and that the parasite is able to promote lymphocyte depletion and defective cytokine response in these tissues.

## Acute *T. cruzi* Infection Induces Expansion of T Cells in SCLN

SCLN are strategically distributed throughout the body, receiving antigens captured from the epidermis and several other epithelia. The most studied SCLN in *T. cruzi* infection are the inguinal, axillary, and brachial. Acute and chronic *T. cruzi* infections promote a significant increase in SCLN size and cell numbers in mice and humans. Studies performed in mice demonstrated that infection leads to increase of cycling cells, which are able to produce IL-2 and proliferate after in vitro activation [44]. Several studies reported polyclonal activation of T/B lymphocytes in SCLN from acutely and chronically infected mice [9]–[11].

Effector T lymphocytes from SCLN of infected mice secrete high levels of IL-4, IL-10, and IFN-γ [44]. These data suggest that T lymphocyte response in SCLN is quite distinct from MLN in acute infection, with increased production of both type-1 and type-2 cytokines, as summarized in Table 1 [44].

T and B lymphocyte apoptosis is also observed in SCLN from infected mice, mainly in the latter stages of infection [13]. Apoptosis in SCLN seems to be induced by AICD, due to control of an extensive state of cell activation. In agreement with this notion, it has been shown that in vivo injection of anti-FasL antibody or zVAD-fmk in acutely infected mice increased the numbers of T/B lymphocytes in SCLN and improved the host immune response to infection [49],[51]. Trans-sialidase seems also to be involved in lymphocyte apoptosis in SCLN from infected mice [30].

Taken together, the data discussed above show that SCLN and MLN lymphocytes are distinctly affected in *T. cruzi* acute infection, with different patterns of T cell activation, proliferation, cytokine responses, and cell death.

## Splenomegaly Is a Constant Feature in *T. cruzi* Infection

**Figure 3:**
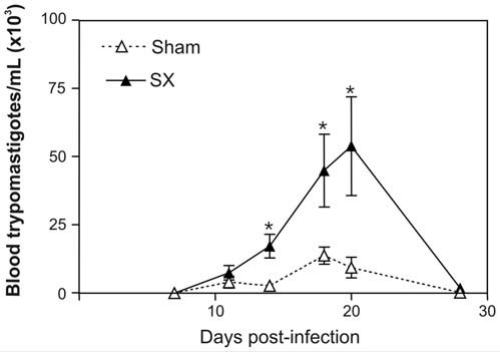
Splenectomy increases host's susceptibility to *T. cruzi* infection. BALB/c mice were submitted to surgery to remove the spleen (SX). Sham-operated mice were used as controls. Ten days after surgery, mice were infected intraperitoneally with 2×10^5^ metacyclic trypomastigote forms of Dm28c clone of *T. cruzi*. Parasitemia was followed during acute phase. In the splenectomized animals, parasitemia was significantly higher, as compared to the sham-operated infected counterparts. Kinetic points with significant differences between SX (*n* = 07, filled line) and sham-operated (*n* = 05, dashed line) groups are indicated. Data were compared by Student's *t* test for independent samples using a Sigma Plot for Windows (version 4.01) package. For parasitemia, data were transformed to parasites/ml for statistical analysis. Data were considered significant if *p* values were <0.05 (*). Data represent mean±standard error. All experiments and animal handling were conducted according to protocols approved by the Oswaldo Cruz Foundation Committee on the Use of Animals.

*T. cruzi* infection promotes splenomegaly in mice and humans. In mice, splenomegaly is observed in both acute and chronic phases of disease. Splenocytes are important cells involved in the host immune response since splenectomy prior to infection increases susceptibility to infection, as ascertained by the numbers of circulating parasites (Figure 3).

Infection promotes spleen T/B lymphocyte activation and expansion. In this respect, trans-sialidase seems to contribute to polyclonal lymphocyte activation and cytokine production by interfering with interaction between dendritic cells and T lymphocytes [52]–[54]. Additionally, other parasite-derived molecules such as racemase and DNA (via Toll-like receptor 9) were proven to induce B cell proliferation [55]–[57].

Although parasite-driven proteins are able to induce B cell expansion, the relative contribution of B cells in acquired resistance upon *T. cruzi* infection remains open to discussion [58]. B cell-deficient mice show increased mortality rates at late stages of infection and a delayed rise in parasitemia, with deficient ability to remove bloodstream trypomastigotes from the circulation [59]–[62]. By contrast, polyclonal B cell activation contributes to the pathological alterations seen in Chagas disease. Antibodies are involved in wasting mice, and auto-reactive antibodies against endocardium and nerves are detected in both mice and humans [63],[64].

Previous data in the literature demonstrated that IL-2 and T lymphocyte proliferation of concanavalin A-stimulated splenocytes from acutely infected mice is depressed [65],[66]. This IL-2 suppression is usually observed in early stages of infection with virulent parasite strains (proportional to stages of high parasite loads) [66],[67]. Activated T lymphocytes in the spleen from *T. cruzi*-infected mice secrete IFN-γ, IL-4, and IL-10, suggesting a type-1 and type-2 mixed profile of cytokine secretion, similar to what is found in SCLN [68]. In humans, co-culture of PBMCs from normal patients with irradiated *T. cruzi* promotes T/B lymphocyte proliferation and secretion of cytokines as IL-1β, IL-2, IL-5, IL-6, IFN-γ, and TNF-α [69].

AICD is observed in spleen-derived activated T and B lymphocytes. It has been shown that Fas selectively kills activated IgG^+^ B lymphocytes specific for parasite antigens [70]. Moreover, CD4^+^ and CD8^+^ T lymphocytes are affected by Fas-induced apoptosis, since activated T lymphocytes increase Fas, FasL expression, and caspase-8 activation in acute infection [51],[68]. Blockade of FasL interaction partially increases CD4^+^ T lymphocyte recovery, in vitro and in vivo [51]. Moreover, in vivo administration of anti-FasL blocked AICD and enhanced T CD8 proliferation in infected mice [71]. Blockade of activated T CD8 death increased IFN-γ secretion in initial stages and IL-4/IL-10 secretion in latter stages of infection [71]. Moreover, it has been shown that IFN-γ is able to promote splenocyte apoptosis by increasing Fas/Fas-L expression, as well as by nitric oxide (NO)-mediated cell death [72]. In fact, NO synthesis in acute *T. cruzi* infection is able to modulate immune response, suppressing T cell proliferation and inducing lymphocyte apoptosis [72]–[74]. In chronic chagasic patients, Fas and TNF-α are also involved in PBMC death and low proliferative capacity of these cells to *T. cruzi* antigens [75].

Lymphocyte apoptosis in *T. cruzi* infection favors the parasite rather than the host, since phagocytic clearance of apoptotic cells increases *T. cruzi* replication inside macrophages [52],[76],[77].

Several groups analyzed the pattern of immune response in the spleen of *T. cruzi*-infected mice. On the other hand, there are few reports on spleen involvement in patients with Chagas disease. In humans, analyses of the spleen are based on chronic patient autopsies. The few data in the literature in humans indicate that spleens from chronic patients are increased in size and weight, with increased areas of lymphoid follicles and thromboembolic phenomena [78],[79].

All together, these data indicate that splenocytes are involved in the control of parasite load in infection. Moreover, processes of polyclonal activation, AICD, and disturbances in cytokine production are also observed in the spleen upon *T. cruzi* infection.

## Concluding Remarks and Perspectives

**Figure 4:**
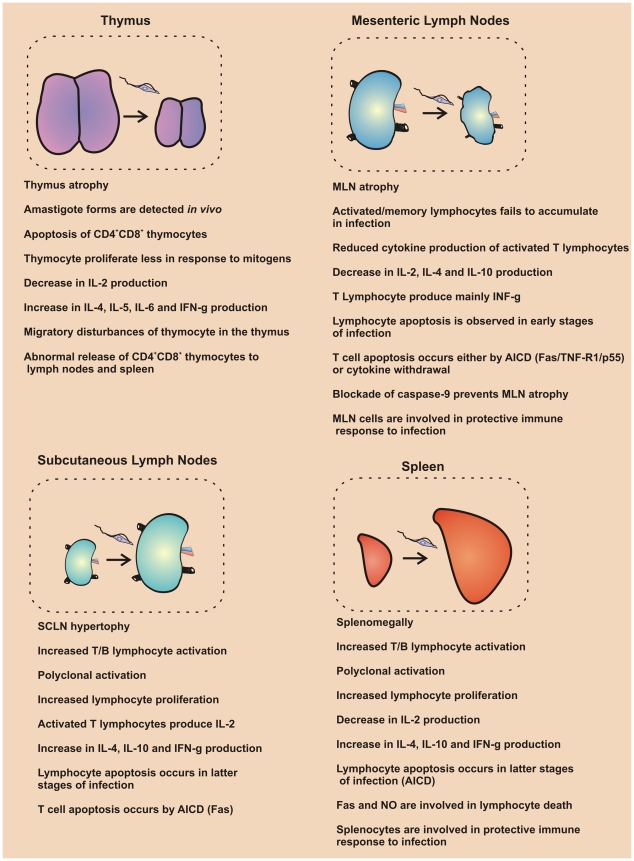
Regional dynamics of immune responses in lymphoid organs following acute experimental *T. cruzi* infection.

*T. cruzi* infection represents a well-documented example of a systemic infectious process. It is usually reported that immune responses mounted in the spleen or in a particular group of lymph nodes represent the immune response of the host. In this review, we unravel the intrinsic importance of specific (and distinct) microenvironments that exist in each lymphoid tissue and propose that immune response in this infection is complex and variable. After conducting a meta-analysis of studies (see Methods in Box 1) performed in the thymus, MLN, SCLN, and spleen, it seems clear that each lymphoid organ has local differences in ratios of lymphocyte expansion that can be related to regional cytokine production and cell death (see Figure 4). Although the reason(s) accounting for these local variations remain unknown, microenvironmental factors are likely involved, including distinct lymphocyte distribution, antigen drainage, or specific types of antigen presenting cells in each lymphoid organ. This actually represents an open and interesting field for future studies.

**Box 1. Methods**

The papers cited in this manuscript were selected based on the following criteria:

1. Stringency of the papers in relation to the subjects discussed
2. High quality of the papers
3. Papers indexed in the PubMed database

The most affected cytokine in acute *T. cruzi* infection is IL-2, an important growth factor for T lymphocytes that is suppressed in the thymus, MLN, and spleen, but not in SCLN. The mechanism involved in IL-2 deprivation in *T. cruzi* infection is still unknown. One possible mechanism involved in IL-2 suppression is the differential distribution of regulatory T cells (T reg cells) among lymphoid organs in infection, since IL-2 could be produced in normal levels and be sequestered by CD25^high^ CD4 T reg cells. In a non-infective model, it was shown that T reg cells consume but do not produce IL-2 [80]–[83]. In fact, previous studies in *T. cruzi* infection demonstrated the presence of suppressive lymphocytes in secondary lymphoid organs [66], and T reg cells were shown to be present in the spleen of infected mice [84]. Moreover, it was recently shown that T regs promote cytokine deprivation-induced apoptosis in T cells [80]. This mechanism might be associated with the thymus and MLN atrophies in infected mice.

It seems conceivable that the differences herein summarized in the regional immune responses to the parasite may play a role in the pathophysiology of Chagas disease, including in the evolution of the tissue lesions. Quantitative differences in the expansion versus apoptosis of regulatory T cells may be relevant candidates in the differential control of tissue lesions in specific sites of the organisms. Also, the cytokine microenvironment that dendritic cells will interact with in a given secondary lymphoid organ will possibly drive, at least partially, its fate in terms of the antigen presenting function. These are open fields for further investigation in both experimental models and in human Chagas disease.

In summary, identifying the factors that drive antigen-specific T lymphocyte expansion and characterizing the mechanisms that result in the cessation of expansion will be relevant to better understanding of the molecular mechanisms underlying regional lymphocyte dynamics secondary to *T. cruzi* infection, which will hopefully contribute to the design of novel immune intervention strategies to control pathology in this unique infection.
